# 140. *Enterococcus faecalis* CL Synthases Have Redundant Roles and Play A Major Role in Phospholipid Redistribution Associated with Daptomycin Resistance

**DOI:** 10.1093/ofid/ofac492.218

**Published:** 2022-12-15

**Authors:** April Nguyen, Vinathi Polamraju, Rutan Zhang, Diana Panesso, Ayesha Khan, Eugenia Mileykovskaya, Truc Cecilia Tran, Libin Xu, Heidi Vitrac, Cesar A Arias

**Affiliations:** University of Texas Health Science Center at Houston, Houston, Texas; University of Texas Health Science Center at Houston, Houston, Texas; University of Washington, Seattle, Washington; Houston Methodist Hospital, Houston, Texas; Clinica Alemana, santiago de chile, Region Metropolitana, Chile; University of Texas Health Science Center at Houston, Houston, Texas; Houston Methodist Research Institute, Houston, Texas; University of Washington, Seattle, Washington; MOBILion Systems, Inc., Chadds Ford, Pennsylvania; Houston Methodist Hospital and Weill Cornell Medical College, Houston, TX

## Abstract

**Background:**

Daptomycin (DAP) is a lipopeptide antibiotic targeting membrane anionic phospholipids (APLs) at the division septum. DAP resistance (DAP-R) has been associated with activation of the LiaFSR system resulting in redistribution of APL microdomains (likely containing cardiolipin, CL) away from the septum. *E. faecalis* (*Efs*) possess two CL synthase genes, (*cls1* and *cls2*) and changes in Cls1 are associated with DAP-R. However, the roles of each enzyme are unknown. Here, we characterize the roles of *cls* genes in DAP-R in the context of LiaFSR activation.

**Methods:**

*cls1* and/or *cls2* were deleted from *Efs* OG117 and OG117Δ*liaX* (DAP-R strain with an activated LiaFSR response). qRT-PCR was used to study gene expression of *cls1* and *cls2* in the *cls* mutants. Membrane lipid content was analyzed using hydrophilic interaction chromatography-mass spectrometry. Mutants were characterized by DAP minimum inhibitory concentration (MIC) using E-test and localization of APL microdomains with 10-N-nonyl-acridine orange.

**Results:**

*cls1* and *cls2* are upregulated in exponential phase of DAP-R *Efs* OG117Δ*liaX* relative to DAP-S *Efs* OG117, with only *cls1* upregulated in stationary phase. Deletion of *cls1* or *cls2* resulted in upregulation of the other *cls* gene, independent of activation of LiaFSR. Lipidomics analysis confirmed that deletion of both *cls* resulted in complete absence of cell membrane CL content. When comparing CL profiles of *Δcls1* relative to *Δcls2* in both DAP-S and DAP-R, both strains produced similar levels and species of CL. However, development of DAP-R caused a change in membrane lipid content, namely, an increase in CL with no significant difference in phosphatidylglycerol compared to DAP-S strain. Evaluation of CL species in DAP-R shows a shift towards species containing longer fatty acid chains and higher saturation. Independent deletions of *cls1* or *cls2* did not revert the DAP phenotype. In contrast, deletion of both *cls* genes decreased the DAP MIC (2-3 fold) relative to the parent strain and restored septal localization of APL microdomains. DAP MIC was restored upon *trans* complementation of either *cls1* or *cls2* into the double deletion mutant.

**Conclusion:**

Our results support a major role of Cls in changes in cell membrane architecture and DAP-R in enterococci, with overlapping roles for Cls1 and Cls2.

**Disclosures:**

**Cesar A. Arias, MD, PhD**, Entasis Phramceuticals: Grant/Research Support|MeMed Diagnostics: Grant/Research Support|Merck: Grant/Research Support.

